# Malnutrition risks and their associated factors among home-living older Chinese adults in Hong Kong: hidden problems in an affluent Chinese community

**DOI:** 10.1186/s12877-019-1148-5

**Published:** 2019-05-23

**Authors:** Martin M. H. Wong, Winnie K. W. So, Kai Chow Choi, Regina Cheung, Helen Y. L. Chan, Janet W. H. Sit, Brenda Ho, Francis Li, Tin Yan Lee, Sek Ying Chair

**Affiliations:** 1 The Nethersole School of Nursing, The Chinese University of Hong Kong, Shatin, New Territories Hong Kong, China; 2 The Neighbourhood Advice-Action Council, North Point, Hong Kong, China

**Keywords:** Malnutrition, Mini nutritional assessment, Community-dwelling older adults, Well-developed society, Chinese

## Abstract

**Background:**

Although China is undergoing rapid economic development, it is facing an ageing population. No data exists on malnutrition risks of older adults in an affluent Chinese society. The aim of this study is to examine these risks and identify their associated factors among home-living older Chinese adults in Hong Kong.

**Methods:**

This is a cross-sectional study, to which home-living subjects aged 60 or above were recruited, between May and September 2017, from a non-governmental community organisation located in three different districts of Hong Kong. Nutritional status was assessed by the Mini Nutritional Assessment (MNA), and its associated factors examined included socio-demographic characteristics, lifestyle, health status and diet. Multivariable logistic regression analysis was performed to identify factors associated with malnutrition risks (MNA < 24).

**Results:**

Six hundred thirteen subjects (mean age: 78.5 ± 7.4; 54.0% females) completed the survey. Nearly 30% (*n* = 179) were at risk of malnutrition. By multivariable logistic regression, subjects (1) whose vision was only fair or unclear, (2) with poor usual appetite and (3) with main meal skipping behaviour had significantly higher malnutrition risk (all *p* < 0.05).

**Conclusions:**

In this affluent Chinese society, the malnutrition risk in older adults is close to the global average, which is a matter for much concern. Interventions are therefore warranted that target vulnerable groups with poor vision, appetite, and meal skipping behaviour.

**Trial registration:**

Not applicable.

## Background

Malnourished older people have poorer functional status [1–3], longer hospital stays [4–7], and increased readmissions [4], morbidity [5] and mortality [4–7]. Early nutritional screening with community interventions would help to identify older adults at risk of malnutrition and improve their nutritional status in a timely manner [8].

Previous literature has identified various risk factors of malnutrition in the community of older adults. Certain socio-demographic characteristics are associated with that risk: older age [9–17], female sex [9, 10, 15, 16, 18, 19], unmarried [16, 20], low education level [10, 12, 16, 18, 19, 21], unemployment [19], low income [18, 21], living alone [12, 19], lifestyle choices including smoking [22] and less physical activity [21], health status including comorbidity [11], the use of dentures [23], chewing difficulty [20, 24, 25] and poor appetite [25, 26]. Although other factors such as alcohol intake [14, 22] and financial support [15, 18, 19] have been investigated, the findings are inconsistent. The relationship between visual or hearing impairment, which is common in older people, and malnutrition are less studied. As for dietary factors, older adults with decreased food intake and fewer meals [13], difficulty in food preparation [27, 28], and less consumption of fruit and vegetables [17, 20, 29, 30], meat [17, 29], milk [30] and other fluids [29] are more prone to malnutrition. However, the relationship between adherence to local dietary guidelines, or dietary behaviour such as meal skipping and food preferences and malnutrition, have been less investigated.

Because of the one-child policy [31], China is facing the problem of an ageing population, with about 30% projected to be older people aged above 60 by 2050 [32]. Among 1.4 billion Chinese, one-fifth of the world’s population, only a few studies exist studying malnutrition [11, 33]. Using Mini Nutritional Assessment (MNA), Han et al. found about 44% of the community Chinese older adults either at risk, or already suffering from malnutrition [11], while Ji et al. identified about 76% of those aged 90 and over were at risk of malnutrition [33]. However, these previous studies were conducted in developing cities. The more affluent cities in China with gross domestic over US$300 billion, such as Hong Kong and other Tier 1 cities, share many similarities with developed countries, such as a smaller family structure and physical inactivity, which may worsen the malnutrition problem. With rapid economic growth in China, a study from an affluent Chinese society is needed to serve as a model for the rapidly developing and ageing future society in mainland China. The aim of this study is therefore to examine the malnutrition risk and identify its associated factors in home-living older Chinese adults in Hong Kong.

## Methods

### Study design and population

This is a cross-sectional survey of the home-living old-age population in Hong Kong. Subjects were recruited through a large registered charitable non-governmental organisation (NGO), in three districts covering nearly one-seventh of the population in Hong Kong. The eligible criteria were (1) aged 60 or above [34], (2) living at home and (3) able to communicate in Chinese. Those with diseases including cognitive impairment were also invited so that the result from this study was representative to the home-living older population where comorbidity is a common issue [35]. By convenience sampling, eligible subjects were contacted by NGO staff by phone for recruitment. Face-to-face interviews were conducted in various community centres or the subjects’ homes by trained NGO social workers and university nursing students from May to September 2017.

### Ethics

This study was conducted according to the Declaration of Helsinki. Ethical approval was obtained from the Survey and Behavioural Research Ethics Committee of the Chinese University of Hong Kong. During the recruitment, eligible subjects were contacted by the NGO staff to ensure confidentiality. They received an information sheet with the details of the study, their rights regarding participation and withdrawal at any stage. They were informed that the survey would be completed anonymously. Those who were interested in participating were requested to sign the consent form. Approval for the use of certain instruments in the study was obtained before data collection.

### Measurement

The survey comprised five sections: nutritional status, socio-demographic characteristics, lifestyle choices, health status and dietary factors.

#### Nutritional status

The MNA was used to assess the global nutritional staus [36], as recommended by the European Society for Clinical Nutrition and Metabolism (ESPEN) [37]. It is an 18-item instrument covering in four sections: anthropometric assessment (weight, height, arm and calf circumference and weight loss), general assessment (lifestyle, medication, stress, mobility, neuro-psychological problems and skin lesion), dietary assessment (number of meals, food and fluid intake, and mode of feeding) and subjective assessment (perceived health and nutritional status) [36]. A booklet with detailed procedures for anthropometric measurement according to the MNA user guide [38] was provided to aid the measurement of the subjects by the interviewers. The MNA score ranges from 0 to 30, with 24–30 points representing normal nutritional status, 17–23.5 representing a risk of malnutrition, and less than 17 points representing malnourishment [36]. The MNA shows good diagnostic ability, with sensitivity of 0.96, specificity of 0.98 and positive predictive value of 0.97, compared with clinical status determined by physician using anthropometric, clinical, biological and dietary parameters [36, 39]. The reliability α was 0.798 in a community-dwelling older Chinese population [11].

#### Socio-demographic characteristics

Data on socio-demographics characteristics were collected: age, sex, marital status, educational level, employment status, monthly household income, receipt of comprehensive social security assistance (CSSA), a financial assistance scheme provided by the Hong Kong government [40], and information on living alone. Lifestyle characteristics, including smoking and drinking status and level of physical activity, were assessed by using the International Physical Activity Questionnaire Short Form (IPAQ-SF), a seven-item instrument measuring the time spent on variously intense forms of physical activity [41]. The Chinese IPAQ-SF was validated in the Hong Kong Chinese population, with an intra-class correlation coefficient of 0.79 and agreement limits of 94% compared with a physical activity log and an MTI accelerometer [42].

#### Health status

Health status was assessed by comorbidity using the Charlson Comorbidity Index (CCI), and other conditions common in older adults such as visual or hearing abilities, use of dentures, difficulty in chewing food and appetite. The presence of any disease was reported by participant who had the condition diagnosed by their physicians. CCI classifies comorbid conditions, with a weighed score of 1,2,3 or 6 assigned to each condition associated with a death risk [43]. The total score of CCI was calculated by the summation of weighed scores of each presented condition of the individual. It was validated in Chinese older adults, with the area under the receiver operating the characteristic curve (AUC) of CCI in predicting one-year mortality of 0.68 [44]. For other common geriatric conditions, they were directly reported or rated in a 3-point scale by subjects to reflect their overall impact to the living of subjects. The question on usual appetite was modified from Council on Nutrition appetite questionnaire [45].

#### Dietary factors

As for dietary factors, the usual consumption of five major food groups (grains, vegetables, fruit, meat and milk) were assessed using culturally specific food frequency list adopted from the Hong Kong Department of Health [46]. Locally standard sizes of bowls, cups and food models were used for clear illustration of the serving size in the interviews. Adherence to the dietary guidelines was determined by comparing the servings of each food group with the recommendations of the Healthy Eating Food Pyramid for the Elderly, developed by the Department of Health [47]. Details of self-cooked food and dietary supplement consumption were obtained in the interviews. Dietary behaviour included favourite food groups, main meal skipping behaviour, and the preferred temperature of food and drink.

### Statistical analysis

Data was presented as means (SD) for continuous variables and frequency (%) for categorical variables. The nutritional status of the participants was dichotomised to (1) normal and (2) at risk or malnourished based on MNA. Socio-demographic and lifestyle characteristics, health status and dietary factors were presented and compared between participants with normal nutritional status (MNA ≥ 24) and at risk or malnourished (MNA < 24) by independent t-test for continuous variables and chi-square test for categorical variables. Binary logistic regression was used to perform univariate analysis of socio-demographic and lifestyle characteristics, health status and dietary factors associated with at-risk or malnourished nutritional status. Those factors with a *p*-value < 0.25 in the univariate analyses were selected as candidate independent variables for backward multivariable logistic regression to identify factors independently associated with at-risk or malnourished status. The results of the final multivariable logistic regression model for the nutritional status outcome were presented by the odds ratios (OR) and their associated 95% confidence intervals (CI) of the significant factors identified. All statistical analyses were performed using IBM SPSS 24.0 (IBM Crop, Armonk, NY). All statistical tests were two-sided with the level of significance set at 0.05.

## Results

**Fig. 1:**
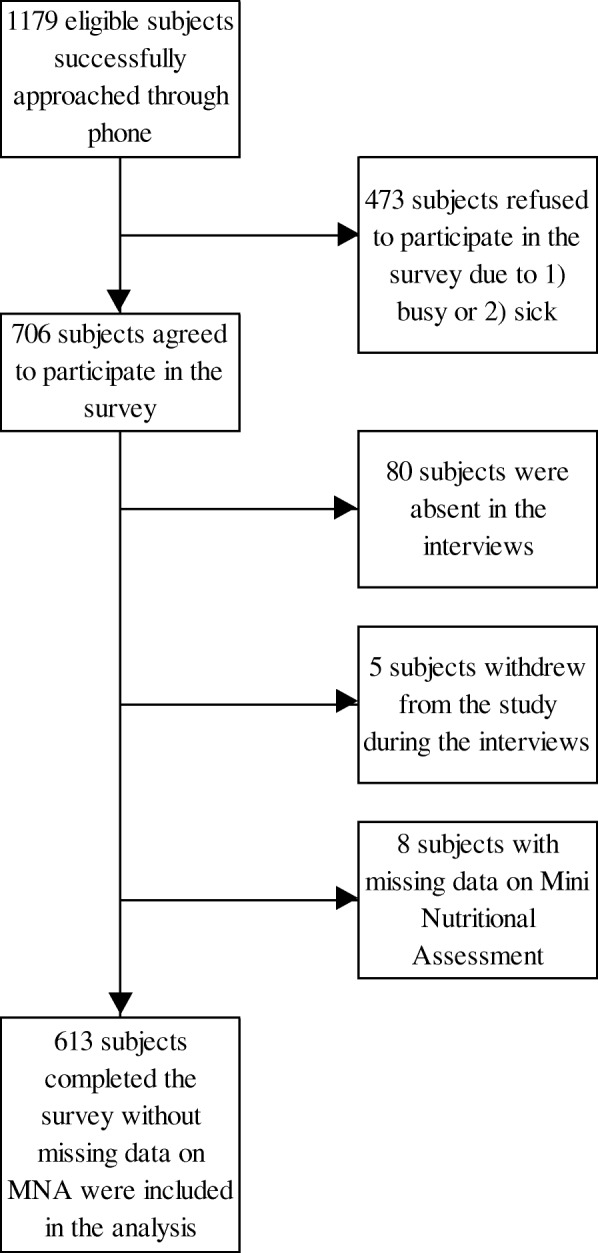
Flowchart of the study. MNA: Mini Nutritional Assessment

A total of 613 subjects completed the survey without missing data on MNA were included in the study (response rate = 52.0%) (Fig. 1). With 54.0% females, the sample collected matched the sex distribution of the Hong Kong older population [48]. The mean age of the subjects was 78.5 ± 7.4, ranging from 60 to 106 (Table 1). The majority had only primary or lower educational attainment (72.8%), were receiving CSSA (59.5%) and living alone (63.3%). About half of the subjects (49.5%) had at least one chronic condition, with total CCI score greater than zero. A considerable proportion reported visual (56.0%) or hearing impairment (41.4%), and more than half reported the use of dentures (63.9%). A large proportion did not adhere to dietary guidelines on the vegetable (82.9%), fruit (72.9%), meat (93.3%) and milk (80.4%) groups, with at least 80% below the recommendations. The majority cooked food for themselves (78.5%) and did not take dietary supplements (63.0%).

**Table 1:** Socio-demographic characteristics, lifestyle characteristics, health status and dietary factors of the participants (*n* = 613)

	Mean (SD) / n (%)			
	All (*n* = 613)	Normal nutritional status (MNA ≥ 24) (*n* = 434)	At risk or malnourished (MNA < 24) (*n* = 179)	*p*-value
Socio-demographic characteristics				
Age (years) ^*^ [range: 60–106]	78.5 (7.4)	78.5 (7.0)	78.6 (8.1)	0.838
Sex^a^				
Male	282 (46.0)	203 (46.8)	79 (44.1)	0.551
Female	331 (54.0)	231 (53.2)	100 (55.9)	
Educational level				
No formal education	188 (30.7)	127 (29.3)	61 (34.1)	0.382
Primary school	258 (42.1)	183 (42.2)	75 (41.9)	
Secondary school or above	167 (27.2)	124 (28.6)	43 (24.0)	
Employment status				
Retired	580 (94.9)	412 (94.9)	168 (94.9)	0.825
Unemployed	18 (2.9)	12 (2.8)	6 (3.4)	
Have part-time/full-time job	13 (2.1)	10 (2.3)	3 (1.7)	
Monthly household income (HK$)				
< 6000	497 (81.1)	352 (81.1)	145 (81.0)	0.992
≥ 6000	64 (10.4)	45 (10.4)	19 (10.6)	
Unsure	49 (8.0)	35 (8.1)	14 (7.8)	
Number of missing	3 (0.5)	2 (0.5)	1 (0.6)	
Received CSSA				
No	247 (40.3)	186 (42.9)	61 (34.1)	0.042
Yes	365 (59.5)	247 (56.9)	118 (65.9)	
Number of missing	1 (0.2)	1 (0.2)	0 (0)	
Marital Status				
Single/divorced/separated/widowed	328 (53.5)	226 (52.1)	102 (57.0)	0.268
Married/cohabited	285 (46.5)	208 (47.9)	77 (43.0)	
Living alone				
No	225 (36.7)	157 (36.2)	68 (38.0)	0.672
Yes	388 (63.3)	277 (63.8)	111 (62.0)	
Lifestyle characteristics				
Smoking status				
Non-smoker	451 (73.6)	319 (73.5)	132 (73.7)	0.055
Ex-smoker	96 (15.7)	75 (17.3)	21 (11.7)	
Current smoker	66 (10.8)	40 (9.2)	26 (14.5)	
Drinking status				
Non-drinker	488 (79.6)	343 (79.0)	145 (81.0)	0.721
Ex-drinker	68 (11.1)	51 (11.8)	17 (9.5)	
Current drinker	57 (9.3)	40 (9.2)	17 (9.5)	
Level of physical activity (IPAQ-SF)				
Low	82 (13.4)	47 (10.8)	35 (19.6)	0.005
Moderate	339 (55.3)	239 (55.1)	100 (55.9)	
High	180 (29.4)	139 (32.0)	41 (22.9)	
Number of missing	12 (2.0)	9 (2.1)	3 (1.7)	
Health status				
Total CCI score^b^				
0	302 (49.3)	221 (50.9)	81 (45.3)	0.059
1	184 (30.0)	134 (30.9)	50 (27.9)	
2	58 (9.5)	34 (7.8)	24 (13.4)	
≥ 3	61 (10.0)	38 (8.8)	23 (12.8)	
Number of missing	8 (1.3)	7 (1.6)	1 (0.6)	
Hearing ability				
Clear	359 (58.6)	273 (62.9)	86 (48.0)	< 0.001
Fair	170 (27.7)	116 (26.7)	54 (30.2)	
Unclear	84 (13.7)	45 (10.4)	39 (21.8)	
Visual ability				
Clear	270 (44.0)	218 (50.2)	52 (29.1)	< 0.001
Fair	198 (32.3)	135 (31.1)	63 (35.2)	
Unclear	145 (23.7)	81 (18.7)	64 (35.8)	
Use of denture				
No	219 (35.7)	160 (36.9)	59 (33.0)	0.373
Yes	392 (63.9)	273 (62.9)	119 (66.5)	
Number of missing	2 (0.3)	1 (0.2)	1 (0.6)	
Difficulty in chewing food				
No	374 (61.0)	276 (63.6)	98 (54.7)	0.038
Yes	238 (38.8)	157 (36.2)	81 (45.3)	
Number of missing	1 (0.2)	1 (0.2)	0 (0)	
Usual appetite				
Good	338 (55.1)	278 (64.1)	60 (33.5)	< 0.001
Normal	241 (39.3)	148 (34.1)	93 (52.0)	
Bad	34 (5.5)	8 (1.8)	26 (14.5)	
Dietary factors				
Below recommendation of the dietary guidelines^c^:				
Grains (3 to 5 bowls per day)	199 (32.5)	127 (29.3)	72 (40.2)	0.008
Vegetables (at least 3 servings per day)	508 (82.9)	351 (80.9)	157 (87.7)	0.041
Fruits (at least 2 servings per day)	447 (72.9)	303 (69.8)	144 (80.4)	0.007
Meats (5 to 6 taels per day)	572 (93.3)	403 (92.9)	169 (94.4)	0.541
Milk (1 to 2 glass per day)	493 (80.4)	352 (81.1)	141 (78.8)	0.473
Usually cooking food myself				
No	127 (20.7)	84 (19.4)	43 (24.0)	0.202
Yes	481 (78.5)	346 (79.7)	135 (75.4)	
Number of missing	5 (0.8)	4 (0.9)	1 (0.6)	
Dietary supplements consumption				
No	386 (63.0)	267 (61.5)	119 (66.5)	0.248
Yes	227 (37.0)	167 (38.5)	60 (33.5)	
Dietary behaviour				
Favour food group				
No preference	54 (8.8)	30 (6.9)	24 (13.4)	0.022
Grains	165 (26.9)	108 (24.9)	57 (31.8)	
Vegetables	167 (27.2)	128 (29.5)	39 (21.8)	
Fruits	70 (11.4)	55 (12.7)	15 (8.4)	
Meats	146 (23.8)	104 (24.0)	42 (23.5)	
Others (milk or fat/salt/sugar)	6 (1.0)	4 (0.9)	2 (1.1)	
Number of missing	5 (0.8)	5 (1.2)	0 (0)	
Usual number of main meals skipped per day				
One or more	106 (17.3)	59 (13.6)	47 (26.3)	< 0.001
None	507 (82.7)	375 (86.4)	132 (73.7)	
Preferred temperature of food and drink				
Warm	365 (59.5)	269 (62.0)	96 (53.6)	0.088
Hot	226 (36.9)	148 (34.1)	78 (43.6)	
Cold	21 (3.4)	16 (3.7)	5 (2.8)	
Number of missing	1 (0.2)	1 (0.2)	0 (0)	

*CCI* Charlson comorbidity index, *CSSA* comprehensive social security assistance, *HK$* Hong Kong dollar, *IPAQ-SF* the international physical activity questionnaire – short form

Data marked with ^*^ are presented as mean (standard deviation), all others are presented as frequency (%)

^a^Sex distribution of the Hong Kong population aged 60 or above: males, 643,258 (47.6%); females, 707,438 (52.4%) [48]

^b^Total score of Charlson comorbidity index was calculated by the summation of weighed scores of each presented condition of the individual

^c^The dietary guidelines were based on the serving size recommendation in the Healthy Eating Food Pyramid for Elderly, developed by Department of Health, Hong Kong [47]. The five major food groups include grains (e.g. rice, noodles, starchy vegetables, bread and oat meals), vegetables (e.g. leafy vegetables, melon, mushroom), fruits (e.g. apple, banana, dried fruits), meats (e.g. beef, fish, egg) and milk (e.g. cow milk, yogurt, cheese) [47]. One bowl equals to 250-300 ml, one serving of vegetable equals to half bowl of cooked vegetables, one serving of fruit equals to one medium-sized fruit, one tael of meats equals to meats with the size of a table tennis ball and one glass equals to 240 ml [47].

**Fig. 2:**
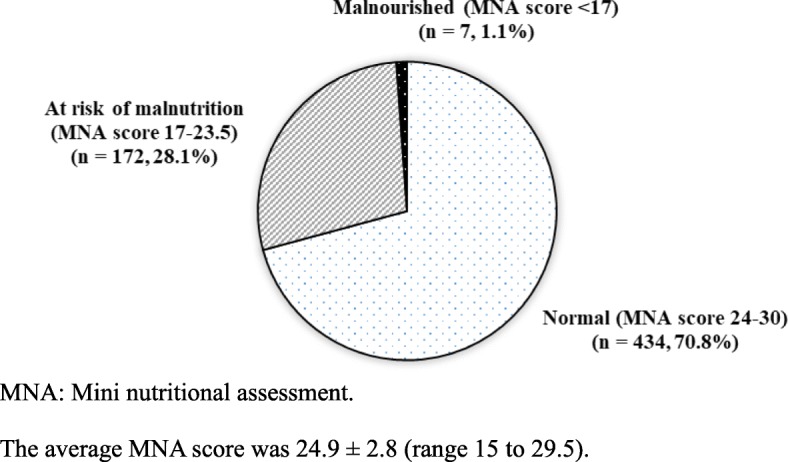
Nutritional status of the participants (*n* = 613). MNA: Mini nutritional assessment. The average MNA score was 24.9 ± 2.8 (range 15 to 29.5)s

The nutritional status of the subjects is shown in Fig. 2. The mean MNA score was 24.9 ± 2.8 and ranged from 15 to 29.5. Nearly 30% of the subjects had MNA below 24, indicating they were either at risk of malnutrition (28.1%) or already malnourished (1.1%). Compared with subjects having normal nutrition status, those who were at risk or malnourished had significantly higher proportion receiving CSSA, poorer visual and hearing ability and usual appetite, more chewing difficulty and main meal skipping behaviour, and different food preference and were less active and below recommendation of the dietary guidelines of grains, vegetables and fruits (all *p* < 0.05) (Table 1).

The results of the univariate analyses of socio-demographic and lifestyle characteristics, health status and dietary factors associated with at risk or malnourished nutritional status are to be found in Table 2. A backward multivariable logistic regression analysis using those factors with *p*-values < 0.25 in the univariate analysis revealed that (1) visual ability, (2) usual appetite and (3) main meal skipping behaviour were significantly and independently associated with at-risk or malnourished status (Table 2). Compared with those with good visual ability, older adults with only fair ability had a higher odds of being at risk or malnourished (adjusted odds ratio (AOR): 1.71, 95% CI: 1.09–2.67, *p* = 0.020) and those with weak ability had an even higher odds (AOR: 2.71, 95% CI: 1.68–4.35, *p* < 0.001). Older adults with a good usual appetite had a decreased odds of being at risk or malnourished (AOR: 0.38, 95% CI: 0.26–0.56, *p* < 0.001), whereas those with little usual appetite had an increased odds (AOR: 4.52, 95% CI: 1.92–10.62, *p* < 0.001), when compared with those who reported a normal usual appetite. Older adults skipping one or more main meals per day had an increased odds of being at risk or malnourished (AOR: 2.03, 95% CI: 1.27–3.25, *p* = 0.003) compared with those without main meal skipping behaviour.

**Table 2:** Factors associated with nutritional status (at risk or malnourished vs normal) (*n* = 613)

	Univariate analysis				Multivariable analysis			
	OR_U_	95% CI		p	OR_A_	95% CI		p
		Lower	Upper			Lower	Upper	
Socio-demographic characteristics								
Received CSSA								
No (ref)	1				NS			
Yes	1.46	1.01	2.09	0.042				
Lifestyle characteristics								
Smoking status								
Non-smoker (ref)	1				NS			
Ex-smoker	0.68	0.40	1.14	0.145				
Current smoker	1.57	0.92	2.68	0.097				
Level of physical activity (IPAQ-SF)								
Low (ref)	1				NS			
Moderate	0.56	0.34	0.92	0.023				
High	0.40	0.23	0.69	0.001				
Health status								
Total CCI score								
0 (ref)	1				NS			
1	1.02	0.67	1.54	0.932				
2	1.93	1.08	3.44	0.027				
≥ 3	1.65	0.93	2.94	0.088				
Hearing ability								
Clear (ref)	1				NS			
Fair	1.48	0.99	2.21	0.058				
Unclear	2.75	1.68	4.50	< 0.001				
Visual ability								
Clear (ref)	1				1			
Fair	1.96	1.28	2.99	0.002	1.71	1.09	2.67	0.020
Unclear	3.31	2.12	5.17	< 0.001	2.71	1.68	4.35	< 0.001
Difficulty in chewing food								
No (ref)	1				NS			
Yes	1.45	1.02	2.07	0.038				
Usual appetite								
Normal (ref)	1				1			
Good	0.34	0.24	0.50	< 0.001	0.38	0.26	0.56	< 0.001
Bad	5.17	2.25	11.91	< 0.001	4.52	1.92	10.62	< 0.001
Dietary factors								
Below recommendation of the dietary guidelines^a^:								
Grains (3 to 5 bowls per day)								
No (ref)	1				NS			
Yes	1.63	1.13	2.34	0.009				
Vegetables (at least 3 servings per day)								
No (ref)	1				NS			
Yes	1.69	1.02	2.80	0.043				
Fruits (at least 2 servings per day)								
No (ref)	1				NS			
Yes	1.78	1.17	2.71	0.008				
Usually cooking food myself								
No (ref)	1				NS			
Yes	0.76	0.50	1.16	0.203				
Dietary supplements consumption								
No (ref)	1				NS			
Yes	0.81	0.56	1.16	0.248				
Dietary behaviour								
Favour food group								
No preference (ref)	1				NS			
Grains	0.66	0.35	1.23	0.192				
Vegetables	0.38	0.20	0.73	0.003				
Fruits	0.34	0.16	0.75	0.007				
Meats	0.50	0.27	0.96	0.038				
Others (milk/fat/salt/sugar)	0.63	0.11	3.71	0.605				
Usual number of main meals skipped per day								
None (ref)	1				1			
One or more	2.26	1.47	3.48	< 0.001	2.03	1.27	3.25	0.003
Preferred temperature of food and drink								
Warm (ref)	1				NS			
Hot	1.48	1.03	2.12	0.034				
Cold	0.88	0.31	2.46	0.801				

*CCI* Charlson comorbidity index, *IPAQ-SF* the international physical activity questionnaire – short form, *NE* not entered into multivariable analysis, *NS* not statistically significant in multivariable analysis, *OR*_*A*_ odds ratio adjusted for other significant factors obtained from backward stepwise logistic regression analysis using variables with *p*-value < 0.25 in univariate analysis as candidate variables, *OR*_*U*_ univariate odds ratio, *ref* Reference group of the categorical variable

^a^The dietary guidelines were based on the serving size recommendation in the Healthy Eating Food Pyramid for Elderly, developed by Department of Health, Hong Kong [47]. The five major food groups include grains (e.g. rice, noodles, starchy vegetables, bread and oat meals), vegetables (e.g. leafy vegetables, melon, mushroom), fruits (e.g. apple, banana, dried fruits), meats (e.g. beef, fish, egg) and milk (e.g. cow milk, yogurt, cheese) [46]. One bowl equals to 250-300 ml, one serving of vegetable equals to half bowl of cooked vegetables, one serving of fruit equals to one medium-sized fruit, one tael of meats equals to meats with the size of a table tennis ball and one glass equals to 240 ml [47]

## Discussion

It has long been believed that malnutrition is an important health issue only in less developed economies where food insecurity and infectious disease prevail [49]. Given the social and economic transformation in China, overweight and obesity have become a research and service focus [50], while malnutrition in vulnerable groups such as older adults have usually been ignored. Our study is the only one to concentrate on malnutrition of older adults in an affluent Chinese community. The findings show that nearly 30% of the subjects were at risk of malnutrition, close to the global average, which revealed that 37.7% of the community older adults were at malnourished risk or already malnourished [51]. This suggests that malnutrition is not limited to developing regions [11, 33], and that more effort should also be put into examining its underlying causes in affluent regions.

By multivariable logistic regression, the high malnutrition risk was found to be associated with (1) fair and poor visual ability, (2) lack of appetite and (3) meal skipping behaviour. Our study found the poorer the visual ability, the higher the odds of being at risk or malnourished. This is consistent with a previous study’s finding that poor vision was associated with higher malnutrition risk among older assisted-living residents [52]. Since poor vision reduces the functional status of older people [53, 54], subjects with impaired sight may find it difficult to feed themselves and go shopping for supplies, causing reduced food intake and thus malnutrition. Previous literature found that older adults having low scores in both basic and instrumental daily living activity [12, 15, 25] had a higher malnutrition risk. Visual problems are common in Hong Kong [55], but the waiting time for new case booking at eye specialist out-patient clinic is extremely long, ranging from 47 to 153 weeks for a stable case [56]. Improvement in eye care services may pose a secondary effect to improve the nutritional status in the visually impaired population.

Our findings on the relationship between poor appetite and high malnutrition risk are consistent with a Netherlands prospective cohort study [26], indicating that one does lead to the other. Poor appetite is associated with lower intake of energy and protein [57], which contributes to malnutrition. A qualitative study on home-living older adults’ views on food reported that the quality of food, including taste and fashion, was of importance [58]. The taste and smell of food can be enhanced using flavourings and seasonings to stimulate appetite [59, 60]. Old-fashioned food and increased food variety might be considered in meal planning for older people [58, 59]. In the affluent Chinese community, the reduced appetite may be caused by depression, especially when the prevalence of depression was high (12.5%) among Hong Kong older adults [61]. This indicated the need to cover multiple dimensions of geriatric problems for nutritional intervention.

In our study, subjects with meal skipping behaviour had a higher malnutrition risk, matching the findings of other studies [10, 13]. Skipping meals may imply insufficient food intake, leading to malnutrition [13]. There are several possible reasons leading to the skipping of meals. First, living alone or being alone in the daytime is widespread in developed regions with smaller family structure [62], increasing the chances of skipping meals as older people prefer not to eat alone [63, 64]. Second, older adults may skip meals because of financial constraints. A comparative study suggest that a pleasant eating environment increases older people’s energy intake at each meal [65]. Canteens for the older adults selling nutritionally balanced meals at low cost may provide a place for them to interact with one another, developing the social support that reduces loneliness and malnutrition risk [66, 67].

The strengths of our study include the use of MNA, which is a well-validated and frequently used nutrition screening tool specifically intended for older adults [36, 51], and the use of materials such as booklets with detailed procedures of anthropometric measurements, and standard sizes of bowls, cups and food models to ensure high reliability during data collection. The limitations of the study include the cross-sectional design, which cannot identify the cause-and-effect relationship between malnutrition and its associated factors, and convenience sampling, which may introduce bias. Furthermore, the local dietary guideline did not consider gender-specific food intake, which may lead to overestimation of insufficient food intake in female requiring relatively less intake than male. However, other gender-specific guidelines such as the Eatwell Guide from UK [68] were not adopted due to cultural and ethnic issues, as Caucasian has larger body size and thus higher nutrition requirement than Chinese. Although a substantial number of comorbid subjects with various diseases were included in the study, some potential subjects declined to participate because of illness, which may have led to lower comorbidity in our sample. As comorbidity is positively associated with malnutrition [11], the implication is that the malnutrition risk reported in our study may be an underestimate.

## Conclusions

To conclude, this study found that a significant proportion of the home-living older adults were at risk of malnutrition in an affluent Chinese community, which causes much concern and deserves attention, revealing a need to examine the impacts of disparity between rich and poor on nutrition in older adults. The results of the multivariable logistic regression analysis found that, fair or poor visual ability, lack of appetite and meal skipping behaviour are associated with high malnutrition risk. Eye care services improvement is vital to reduce the problem of visual impairment and thus malnutrition. The sensory perception of flavour, wide variety and traditional types of food can improve the appetite of older adults. Older people’s canteens can be developed to allow interaction among older adults to enhance social support, while providing nutritionally balanced meals at low cost.

## Abbreviations

AOR: Adjusted odds ratio
AUC: Area under the receiver operating the characteristic curve
CCI: Charlson Comorbidity Index
CI: Confidence interval
CSSA: Comprehensive social security assistance
ESPEN: European Society for Clinical Nutrition and Metabolism
HK$: Hong Kong dollar
IPAQ-SF: International Physical Activity Questionnaire Short Form
MNA: Mini Nutritional Assessment
NE: Not entered into multivariable analysis
NGO: Non-governmental organisation
NS: Not statistically significant in multivariable analysis
OR: Odds ratios
OR_A_: Odds ratio adjusted for other significant factors obtained from backward stepwise logistic regression analysis using variables with *p*-value < 0.25 in univariate analysis as candidate variables
OR_U_: Univariate odds ratio
ref.: Reference group of the categorical variable
SD: Standard deviation
UK: United Kingdom
